# Fabrication of ordered honeycomb porous poly(vinyl chloride) thin film doped with a Schiff base and nickel(II) chloride

**DOI:** 10.1016/j.heliyon.2018.e00743

**Published:** 2018-08-18

**Authors:** Hassan Hashim, Gamal A. El-Hiti, Mohammad Hayal Alotaibi, Dina S. Ahmed, Emad Yousif

**Affiliations:** a Department of Physics, College of Science, Al-Nahrain University, Baghdad, Iraq; b Department of Optometry, College of Applied Medical Sciences, King Saud University, P.O. Box 10219, Riyadh 11433, Saudi Arabia; c National Center for Petrochemicals Technology, King Abdulaziz City for Science and Technology, P.O. Box 6086, Riyadh 11442, Saudi Arabia; d Department of Chemistry, College of Science, Al-Nahrain University, Baghdad 64021, Iraq

**Keywords:** Materials chemistry

## Abstract

A modified poly(vinyl chloride) honeycomb thin film containing a low concentration of a thiadiazole Schiff base and nickel(II) chloride was successfully fabricated using the casting process. The surface morphology of the synthesized thin film was investigated using the scanning electronic microscopy. The synthesized poly(vinyl chloride) thin film was found to have a homogeneous surface morphology with a high crystalline nature. The addition of nickel(II) chloride was discovered to be vital for the formation of the honeycomb like structure.

## 1 Introduction

Physical and chemical properties of polymeric materials determine their utilization [1]. For commercial uses, the polymeric materials should have good physical and mechanical properties, low cost production, high stability and low specific gravity [2, 3]. The surface morphology of polymeric materials can be controlled by various parameters such as temperature [4], concentration [5], film thickness and structure [6] and dopant concentration. Poly(vinyl chloride), PVC, has various commercial and industrial applications [7]. However, despite its advantages, it bears poor thermal stability and a low impact strength [8, 9]. Therefore, attention has been paid to modify the physical and chemical properties of PVC for long term use [10, 11, 12, 13, 14, 15, 16, 17, 18, 19]. The most common routes for the modification process involves chemical (grafting copolymerization) and physical (blending) modifications [20, 21].

Nanometer and micrometer highly ordered polymeric films can be used in cell culture, batteries and optical devices [22, 23]. Porous films with highly order structures have various applications and can be used in patterned templates [24] photonic crystals [25] sensors [26], optical technology [27], catalysis [28] and membrane separation [29]. Honeycomb structures have unique characteristics such as high stability, low density, excellent mechanical properties and large surface area [30, 31]. Several approaches have been reported for the fabrication of honeycomb materials in which particles sizes were controlled, but some of these processes require expensive multiple steps [32, 33, 34, 35, 36, 37, 38, 39]. Some development in the preparation of honeycomb patterned film has been made using the star-shaped or branched polymers. However, their molecular weight and branching degree were generally low [40]. Due to the highly remarkable lightweight of honeycomb materials, we became interested in their production. The current work deals with the fabrication of highly ordered honeycomb PVC thin film doped with a Schiff base and nickel(II) chloride using the casting method. The structure of PVC thin film produced was characterized by the scanning electronic microscopy (SEM) and energy-dispersive X-ray spectroscopy (EDX).

## 2 Experimental

**Fig. 1:**
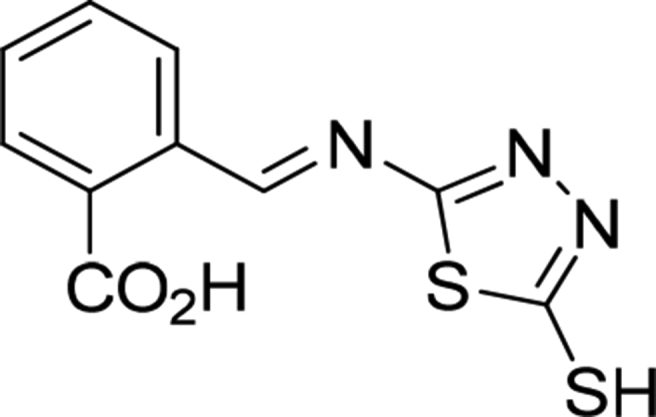
Structural formula of the Schiff base used.

Schiff base (Fig. 1) was synthesized, as a white solid in 83% yield, as previously reported from the reaction of an equimolar mixture of 5-amino-1,3,4-thiadiazole-2-thiol and 2-carboxybenzaldehyde in refluxing ethanol for 2.5 h in the presence of acetic acid as a catalyst [41]. The structure of the Schiff base was confirmed and its spectroscopic data were consistent with those reported [42]. The synthesized thin films were structurally characterized by the SEM using Inspect S50 microscope (FEI Company, Czechia, Czech Republic) at an accelerating voltage of 15 Kv. The EDX measurements wear carried out on Bruker XFlash^®^ 6 10 (Bruker, Tokyo, Japan).

Ethanol was added in a dropwise manner the commercial PVC solution in tetrahydrofuran and the solid formed was collected by filtration and dried under vacuum at room temperature for 24 h. A mixture of PVC (1.0 g) and Schiff base (0.265 g) in tetrahydrofuran (THF; 150 mL) was refluxed for 3 h. A solution of nickel chloride tetrahydrate (NiCl_2_.4H_2_O; 0.3 g) in THF (5 mL) was added and the whole mixture was refluxed for 3 h. A glass plate that contains 15 holes (4 × 4 cm^2^) was washed with THF several times and dried at room temperature. The PVC solution was casted onto the clean glass plate and dried at room temperature for 24 h. The samples were dried further under reduced pressure at 25 °C for 3 h to ensure the removal of any residual solvent left. The films were removed from the glass plate and their thickness (*ca*. 40 μm) was measured using a Digital Caliper DIN 862 micrometer (Vogel GmbH, Kevelaer, Germany). The micrometer has a reading error of *ca*. 0.01 mm. Aluminum plate stands with a thickness of 0.6 mm (Q-Panel Company, Homestead, FL, USA) were used for the fixation of the PVC films.

## 3 Results and discussion

**Fig. 2:**
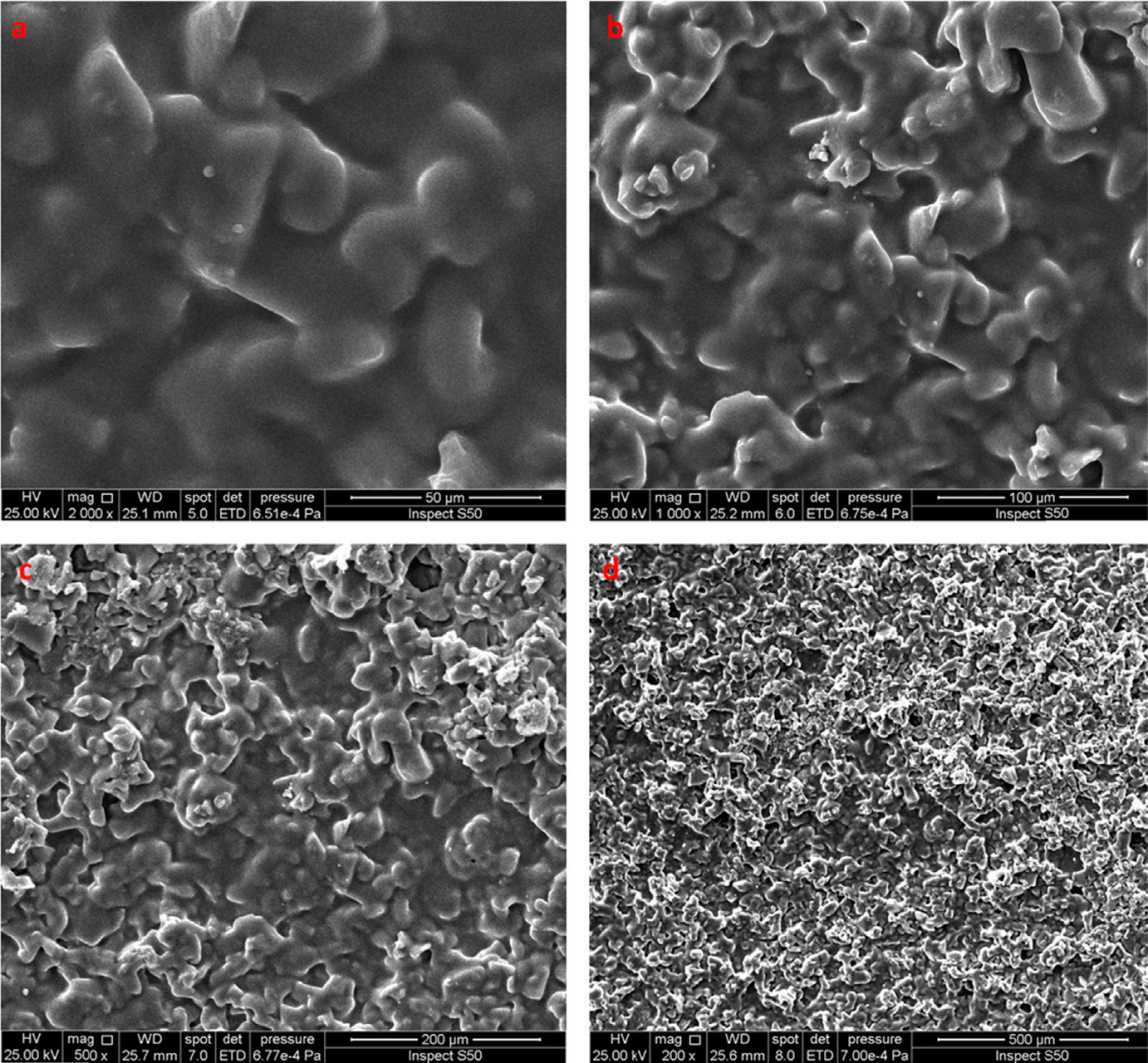
The SEM images of PVC film containing Schiff base: (a) 50 μm; (b) 100 μm; (c) 200 μm; (d) 500 μm.

The SEM is usually used to assist the compatibility level between polymeric film various components in which phase separations and interfaces could be detected. The compatibility level within the polymer matrix and dopants can influence the ionic conductivity, thermal and mechanical properties of the polymeric films [43]. Also, the SEM topography gives an indication for the size and shape of the particles. The surface morphology of PVC film containing Schiff base was inspected by the SEM at various magnification (Fig. 2). The SEM images of PVC film indicated a smooth surface that confirms satisfactory homogeneity and miscibility between the polymer matrix and Schiff base.

**Fig. 3:**
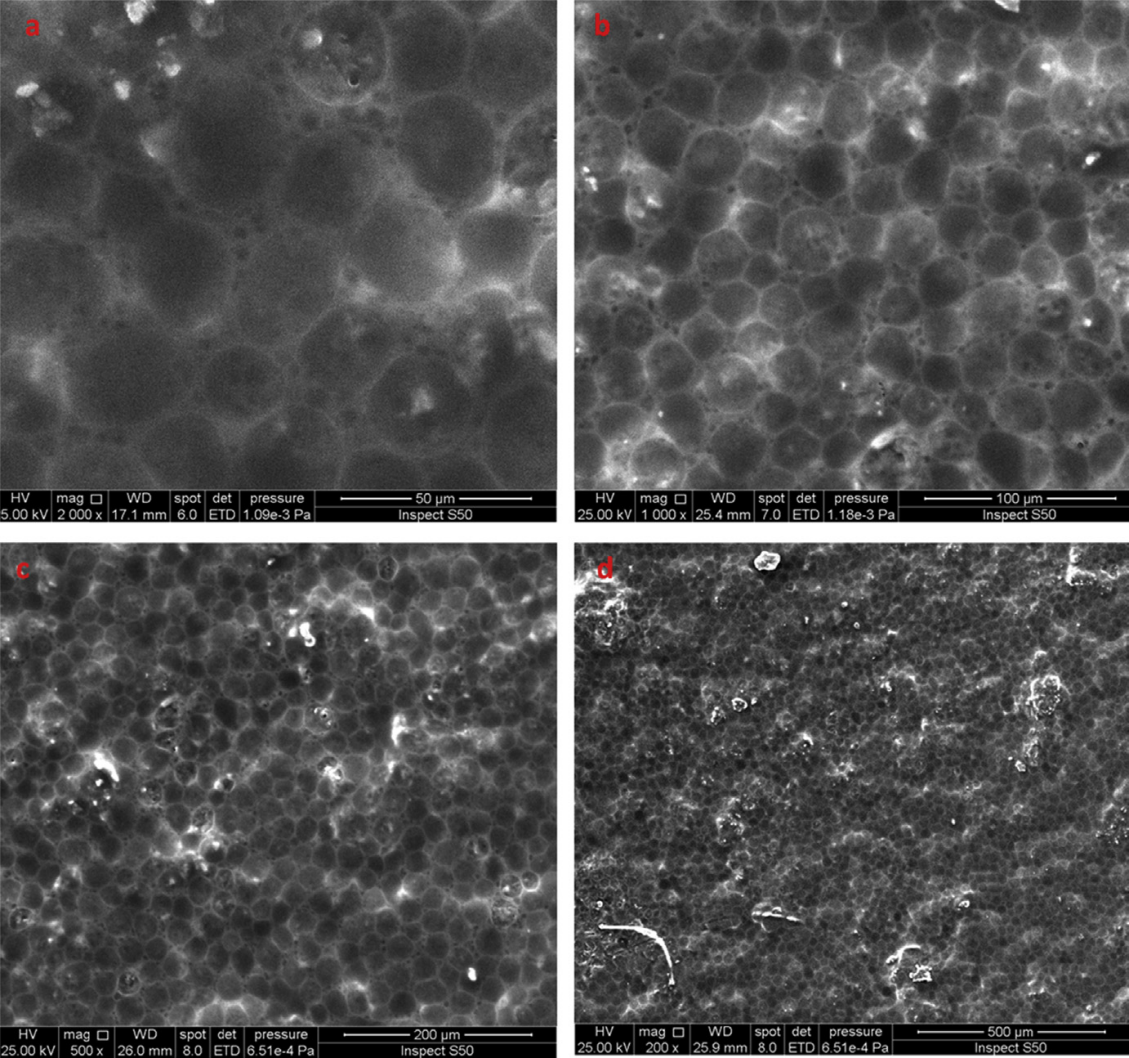
The SEM images of PVC film containing Schiff base and Ni(II) chloride: (a) 50 μm; (b) 100 μm; (c) 200 μm; (d) 500 μm.

Nickel(II) chloride was added to the PVC containing Schiff base and the morphology of the produced film was inspected by the SEM. Fig. 3 indicated that the surface morphology of the PVC film has changed dramatically and turned to be rough with homogeneous composite matrix and phase separation. In addition, the images displayed the honeycomb-like shape morphology of the PVC film The rough surface could be due to the interaction or coordination between the PVC-Schiff base blend and Ni(II) ion as a result of cross–linking. The images indicated that the PVC blend was compatible with a uniform matrix [44]. Evidently, both Schiff base and NiCl_2_ were regularly distributed within the PVC chains (Fig. 3). A number of spherical pores were observed on the surface possibly as a result of the rapid evaporation of tetrahydrofuran used as a solvent in film preparation. The pore size was varying as a result of the difference in the driving force for the phase separation [45]. It is believed that the Ni–Schiff base complex involves coordination between Ni(II) atom and two molecules of the ligand. The Ni atom has a square planar arrangement and coordinates to the imine nitrogen atom and the oxygen atom of the carboxyl group of the ligand. Similar observations have been reported [46, 47].

The morphology of the PVC sample doped with Ni^2+^ ions has changed to the hexagonal shape. The hexagonal shape and the Ni^2+^ ion could act as short-term stabilizers which can enhance the PVC stability significantly [48]. On the other hand, the OH moiety within the carboxyl group of the Schiff base could act as a long-term stabilizer. It can be concluded that, the SEM study reveals that the synthesized PVC film containing Schiff base and NiCl_2_ was highly porous in nature. Such porous structure could be attributed to the coordination and incorporation of the Ni ions on the surface and mostly within the polymer matrix. Generally, the porous nature of the film means a large surface area and a small crystalline size [49]. It has been reported that the structure of the honeycomb is highly dependent on various factors such as solvent type, polymer side-chain length and the polymer concentration [49]. The SEM topography has been used as a powerful tool to detect both the size and shape of particles [50, 51, 52, 53].

**Fig. 4:**
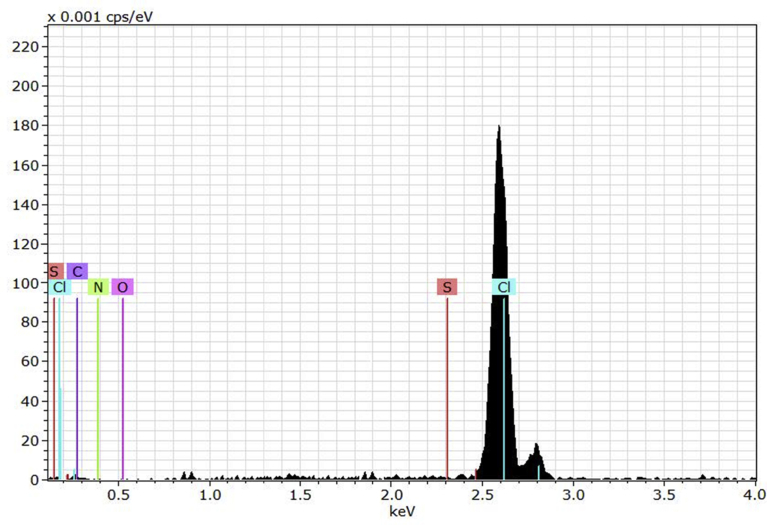
EDX spectrum of PVC film containing Schiff base.

The EDX technique can be used along with the SEM to detect the structure of the polymeric materials. The EDX can detect the elemental composition of the synthesized PVC film [54]. Fig. 4 showed the EDX pattern for the synthesized the PVC film containing Schiff base in the absence of nickel(II) ions. Cleary, it indicated the presence of the elements for both PVC and Schiff base.

**Fig. 5:**
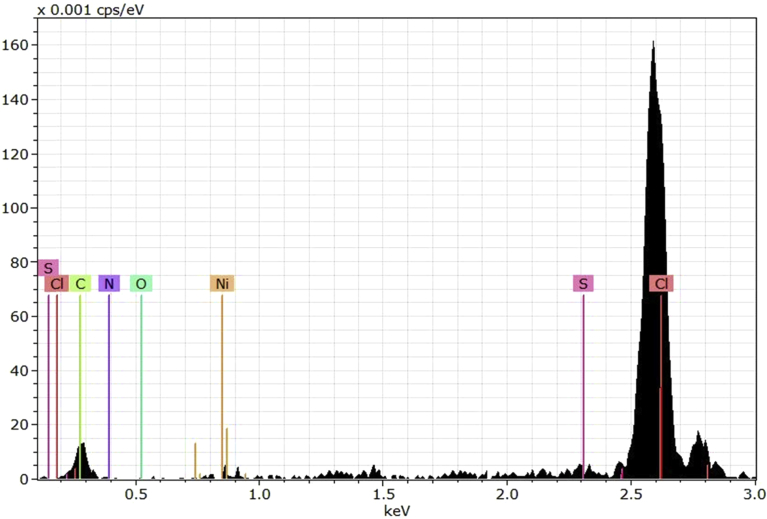
EDX spectrum of PVC film containing Schiff base and Ni(II) chloride.

The EDX patterns for the PVC film containing both Schiff base and nickel(II) chloride is shown in Fig. 5. Evidently, the spectrum showed a very strong abundance for the chlorine which is a strong evidence that the NiCl_2_ are incorporated with the PVC matrix. It shows the presence of a new band that is corresponding to Ni. The assignments of the EDX peaks were in agreement with the literatures [55, 56, 57, 58].

## 4 Conclusions

A highly ordered honeycomb PVC thin film doped with a low concentration of a Schiff base and nickel(II) chloride was synthesized by the casting method. The scanning electronic microscopy indicated that the synthesized PVC film has a honeycomb uniform surface morphology with different particle size. In addition, the energy-dispersive X-ray spectroscopy showed evidence for the incorporation of the nickel(II) chloride within the PVC matrix. It has been proven that the presence of nickel(II) chloride is the main reason for the fabrication of honeycomb PVC structure. The process proposed is increasing, simple and could have potential to be used to produce PVC honeycomb thin films in a commercial scale. However, a more detailed study is needed to understand better the role of nickel(II) chloride and any other Lewis acids on the fabrication of honeycomb PVC like structure.

## Declarations

### Author contribution statement

Hassan Hashim, Gamal A. El-Hiti, Emad Yousif: Conceived and designed the experiments; Wrote the paper.

Mohammad Hayal Alotaibi: Contributed reagents, materials, analysis tools or data; Wrote the paper.

Dina S. Ahmed: Performed the experiments; Analyzed and interpreted the data.

### Funding statement

This work was supported by King Abdulaziz City for Science and Technology (KACST), Saudi Arabia (grant No. 20-0180) and Al-Nahrain University.

### Competing interest statement

The authors declare no conflict of interest.

### Additional information

No additional information is available for this paper.

## References

[1] Su W.-F. (2003). Chemical and physical properties of polymers.

[2] Namazi H. (2017). Polymers in our daily life. Bioimpacts.

[3] Huang H., Talreja R. (2006). Numerical simulation of matrix micro-cracking in short fiber reinforced polymer composites: initiation and propagation. Compos. Sci. Technol..

[4] Bendjedidi H., Attaf A., Saidi H., Aida M.S., Semmari S., Bouhdjar A., Benkhetta Y. (2015). Properties of n-type SnO_2_ semiconductor prepared by spray ultrasonic technique for photovoltaic applications. J. Semicond..

[5] Abdelkrim A., Rahmane S., Abdelouahab O., Abdelmalek N., Brahim G. (2016). Effect of solution concentration on the structural, optical and electrical properties of SnO_2_ thin films prepared by spray pyrolysis. Optik.

[6] Abdallah B., Kakhia M., Abou Shaker S. (2016). Deposition of Na_2_WO_4_ films by ultrasonic spray pyrolysis: effect of thickness on the crystallographic and sensing properties. Compos. Interfaces.

[7] Allsopp M.W., Vianello G. (2012). Poly(Vinyl chloride). Ullmann's Encyclopedia of Industrial Chemistry.

[8] Cooray B., Scott G. (1980). The effect of thermal processing on PVC–VI. The role of hydrogen chloride. Eur. Polym. J..

[9] Jellinek H.H.G. (1978). Aspects of Degradation and Stabilization of Polymers.

[10] Abdelrazek E.M., Elashmawi I.S. (2008). Characterization and physical properties of CoCl_2_ filled polyethyl-methacrylate films. Polym. Compos..

[11] Cadogan D.F., Howick C.J. (2000). Plasticizers. Ullmann's Encyclopedia of Industrial Chemistry.

[12] Ghazi D., El-Hiti G.A., Yousif E., Ahmed D.S., Alotaibi M.H. (2018). The effect of ultraviolet irradiation on the physicochemical properties of poly(vinyl chloride) films containing organotin(IV) complexes as photostabilizers. Molecules.

[13] Ahmed D.S., El-Hiti G.A., Hameed A.S., Yousif E., Ahmed A. (2017). New tetra-Schiff bases as efficient photostabilizers for poly(vinyl chloride). Molecules.

[14] Ali M.M., El-Hiti G.A., Yousif E. (2016). Photostabilizng efficiency of poly(vinyl chloride) in the presence of organotin(IV) complexes as photostabilizers. Molecules.

[15] Yousif E., Hasan A., El-Hiti G.A. (2016). Spectroscopic, physical and topography of photochemical process of PVC films in the presence of Schiff base metal complexes. Polymers.

[16] Yousif E., El-Hiti G.A., Hussain Z., Altaie A. (2015). Viscoelastic, spectroscopic and microscopic study of the photo irradiation effect on the stability of PVC in the presence of sulfamethoxazole Schiff's bases. Polymers.

[17] Balakit A.A., Ahmed A., El-Hiti G.A., Smith K., Yousif E. (2015). Synthesis of new thiophene derivatives and their use as photostabilizers for rigid poly(vinyl chloride). Int. J. Polym. Sci..

[18] Jia P., Zhang M., Hu L., Wang R., Sun C., Zhou Y. (2017). Cardanol groups grafted on poly(vinyl chloride)—synthesis, performance and plasticization mechanism. Polymers.

[19] Jia P., Feng G., Boa C., Hu L., Yang X., Zhang L., Zhang M., Zhou Y. (2018). A composition of phosphaphenanthrene groups-containing castor-oil -based phosphate plasticizer for PVC: synthesis, characterization and property. J. Ind. Eng. Chem..

[20] Wang C., Wang H., Fu J., Gu G. (2014). Effects of additives on PVC plastics surface and the natural flotability. Colloid Surf. A.

[21] Hasan M., Banerjee A.N., Lee M. (2015). Enhanced thermo-optical performance and high BET surface area of graphene@PVC nanocomposite fibers prepared by simple facile deposition technique: N_2_ adsorption study. J. Ind. Eng. Chem..

[22] Galeotti F., Andicsova A., Yunus S., Botta C. (2012). Precise surface patterning of silk fibroin films by breath figures. Soft Matter.

[23] Du C., Zhang A.J., Bai H., Li L. (2013). Robust microsieves with excellent solvent resistance: cross-linkage of perforated polymer films with honeycomb structure. ACS Macro Lett..

[24] Connal L.A., Qiao G.G. (2006). Preparation of porous poly(dimethylsiloxane)-based honeycomb materials with hierarchal surface features and their use as soft-lithography templates. Adv. Mater..

[25] Noda S., Chutinan A., Imada M. (2000). Trapping and emission of photons by a single defect in a photonic bandgap structure. Nature.

[26] Liang L., Ma Y., Sims S., Wu L. (2015). A patterned porous polymer film for localized capture of insulin and glucose-responsive release. J. Mater. Chem. B.

[27] Yabu H., Shimomura M. (2005). Simple fabrication of micro lens arrays. Langmuir.

[28] Tanev P.T., Chibwe M., Pinnavaia T.J. (1994). Titanium-containing mesoporous molecular sieves for catalytic oxidation of aromatic compounds. Nature.

[29] Gugliuzza A., Aceto M.C., Macedonio F., Drioli E. (2008). Water droplets as template for next-generation self-assembled poly-(etheretherketone) with cardo membranes. Phys. Chem. B.

[30] Toyoda M., Sakagami K., Takahashi D., Morimoto M. (2011). Effect of a honeycomb on the sound absorption characteristics of panel-type absorbers. Appl. Acoust..

[31] Chen J., Tuo W., Zhang X., He C., Xie J., Liu C. (2016). Compressive failure modes and parameter optimization of the trabecular structure of biomimetic fully integrated honeycomb plates. Mater. Sci. Eng. C.

[32] Li M., Xu S., Kumacheva E. (2000). Convection in polymeric fluids subjected to vertical temperature gradients. Macromoleculs.

[33] Srinivasarao M., Collings D., Philips A., Patel S. (2001). Three-dimensionally ordered array of air bubbles in a polymer film. Science.

[34] Peng J., Han Y., Fu J., Yang Y., Li B. (2003). Formation of regular hole pattern in polymer films. Macromol. Chem. Phys..

[35] Bolognesi A., Mercogliano C., Yunus S. (2005). Self-organization of polystyrenes into ordered microstructured films and their replication by soft lithography. Langmuir.

[36] Heng L., Wang B., Li M., Zhang Y., Jiang L. (2013). Advances in fabrication materials of honeycomb structure films by the breath-figure method. Materials.

[37] Dou Y., Jin M., Zhou G., Shui L. (2015). Breath figure method for construction of honeycomb films. Membranes.

[38] Liu C.-X., Lang W.-Z., Shi B.-B., Guo Y.-J. (2013). Fabrication of ordered honeycomb porous polyvinyl chloride (PVC) films by breath figures method. Mater. Lett..

[39] Zhang A., Bai H., Li L. (2015). Breath figure: a nature-inspired preparation method for ordered porous films. Chem. Rev..

[40] Qiang X., Ma X., Li Z., Hou X. (2014). Synthesis of star-shaped polyhedral oligomeric silsesquioxane (POSS) fluorinated acrylates for hydrophobic honeycomb porous film application. Colloid Polym. Sci..

[41] Shaalan N., Laftah N., Muslih R., Yousif E. (2016). Photostability study of some modified poly(vinyl chloride) containing pendant Schiff's bases. Baghdad Sci. J..

[42] Shaalan N., Laftah N., El-Hiti G.A., Alotaibi M.H., Muslih R., Ahmed D.S., Yousif E. (2018). Poly(vinyl chloride) photostabilization in the presence of Schiff bases containing a thiadiazole moiety. Molecules.

[43] Kayyarapu B., Kumar M.Y., Mohommad H.B., Neeruganti G.O., Chekuri R. (2016). Structural, thermal and optical properties of pure and Mn^2+^ doped poly(vinyl chloride) films. Mater. Res..

[44] Wu T.-M., Lin Y.-W., Liao C.-S. (2005). Preparation and characterization of polyaniline/multi–walled carbon nanotube composites. Carbon.

[45] Rhoo H.-J., Kim H.-T., Park J.K., Hwang T.-S. (1997). Ionic conduction in plasticized PVCPMMA blend polymer electrolytes. Electrochim. Acta.

[46] Sun W.-H., Wu L.-L., Ye L., Xin Y., Zhang Y., Liu H., Lv K.-W., Shang C.-N., You Z., Li W. (2017). Two mononuclear nickel(II) complexes with Schiff base ligands: synthesis, crystal structures and antibacterial activities. Inorg. Nano Metal Chem..

[47] Ourari A., Bougossa I., Bouacida S., Aggoun D., Ruiz-Rosas R., Morallon E., Merazig H. (2017). Synthesis, characterization and X-ray crystal structure of novel nickel Schiff base complexes and investigation of their catalytic activity in the electrocatalytic reduction of alkyl and aryl halides. J. Iran. Chem. Soc..

[48] Rahman M.Y.A., Ahmad A., Lee T.K., Farina Y., Dahlan H.D. (2011). Effect of ethylene carbonate(EC) plasticizer on poly(vinyl chloride)-liquid 50% epoxidised natural rubber (LENR50) based polymer electrolyte. Mater. Sci. Appl..

[49] Huh M., Gauthier M., Yun S. (2016). Honeycomb structured porous films prepared from arborescent graft polystyrenes via the breath figures method. Polymer.

[50] Ansari F., Nazari P., Payandeh M., Asl F.M., Abdollahi-Nejand B., Ahmadi V., Taghiloo J., Salavati-Niasari M. (2018). Novel nanostructured electron transport compact layer for efficient and large-area perovskite solar cells using acidic treatment of titanium layer. Nanotechnology.

[51] Amiri O., Salavati-Niasari M., Mir N., Beshkar F., Saadat M., Ansari F. (2018). Plasmonic enhancement of dye-sensitized solar cells by using Au-decorated Ag dendrites as a morphology-engineered. Renew. Energy.

[52] Chiuzbăian S.G., Brignolo S., Hague C.F., Delaunay R., Guarise M., Nicolaou A., Yang Z., Zhou H., Mariot J.-M. (2017). Spectroscopic evidence for superexchange in the ferromagnetic spinel FeCr_2_S_4_. J. Phys. Chem. C.

[53] Pei Y., Duan C. (2017). Study on stress-wave propagation and residual stress distribution of Ti-17 titanium alloy by laser shock peening. J. Appl. Phys..

[54] Wang Z.M., Wagner J., Ghosal S., Bedi G., Wall S. (2017). SEM/EDS and optical microscopy analyses of microplastics in ocean trawl and fish guts. Sci. Total Environ..

[55] Tooma M.A., Najim T.S., Alsalhy Q.F., Marino T., Criscuoli A., Giorno L., Figoli A. (2015). Modification of polyvinyl chloride (PVC) membrane for vacuum membrane distillation (VMD) application. Desalination.

[56] Barakat A., Al-NoaimiM, Suleiman M., Aldwayyan A.S., Hammouti B., Ben Hadda T., Haddad S.F., Boshaala A., Warad I. (2013). One step synthesis of NiO nanoparticles via solid-state thermal decomposition at low-temperature of novel aqua(2,9-dimethyl-1,10-phenanthroline)NiCl_2_ complex. Int. J. Mol. Sci..

[57] Hashem M., Saion E., Al-Hada N.M., Kamari H.M., Shaari A.H., Talib Z.A., Paiman S.B., Kamarudeen M.A. (2016). Fabrication and characterization of semiconductor nickel oxide (NiO) nanoparticles manufactured using a facile thermal treatment. Results Phys..

[58] Gao S., Yang L., Deng B., Zhang J. (2017). Corrosion mechanism for local enrichment of acids and copper ions in copper-insulating paper contacts leading to the acceleration of copper sulfide formation induced by dibenzyl disulfide. RSC Adv..

